# Complete Genome Sequence of Sinorhizobium meliloti S35m, a Salt-Tolerant Isolate from Alfalfa Rhizosphere in Soil Native to the Caucasus Region

**DOI:** 10.1128/MRA.01417-20

**Published:** 2021-03-18

**Authors:** Victoria S. Muntyan, Alexey M. Afonin, Maria E. Vladimirova, Alla S. Saksaganskaya, Emma S. Gribchenko, Olga Baturina, Marina L. Roumiantseva

**Affiliations:** a All-Russia Research Institute for Agricultural Microbiology (ARRIAM), Laboratory of Genetics and Breeding of Microorganisms, Saint Petersburg, Russia; b All-Russia Research Institute for Agricultural Microbiology (ARRIAM), Laboratory of Genetics of Plant-Microbe Interactions, Saint Petersburg, Russia; c Institute of Chemical Biology and Fundamental Medicine, Siberian Branch of the Russian Academy of Sciences (ICBFM SB RAS), Novosibirsk, Russia; University of Arizona

## Abstract

The genome of a symbiotically effective salt-tolerant strain, Sinorhizobium meliloti S35m, isolated from alfalfa rhizosphere in soil native to the Caucasus region, was sequenced. Genomic islands, prophages, and elements of a potential CRISPR/Cas I type (Cas3_0_I) system were identified in the genome.

## Abstract (precis)

The genome of a symbiotically effective salt-tolerant strain, Sinorhizobium meliloti S35m, isolated from alfalfa rhizosphere in soil native to the Caucasian region, was sequenced. Genomic islands, prophages, and elements of a potential CRISPR/Cas I type (Cas3_0_I) system were identified in the genome.

## ANNOUNCEMENT

The strain Sinorhizobium meliloti S35m was isolated from a root nodule of Medicago sativa subsp. *varia* (Martyn) Arcang. This nodule was formed on the roots of an alfalfa plant grown from a surface-sterilized seed (method from reference 1) inoculated with water extract from alfalfa rhizosphere in a soil sample from the Caucasus region (2, 3). The strain can grow on tryptone-yeast extract (TY) medium (4) with up to 0.75 M NaCl (5) and forms effective symbiosis with *Medicago sativa* subsp. *varia* var. “Vega 87” (6). The exact mechanisms behind the salt tolerance of this strain are still not fully understood.

This strain was stored in TY medium containing 20% (vol/vol) glycerol at −70°C. A single colony of S35m was grown overnight in TY broth (28°C, 180 rpm shaking). Genomic DNA (gDNA) was isolated using the phenol-chloroform extraction method and used for both the Nanopore and Illumina sequencing (7). To obtain fragments of about 600 bp, 1 μg of gDNA was sheared in a microTUBE AFA fiber snap-cap tube Covaris S2 system. The paired-end library was produced using a NEBNext Ultra II DNA library prep kit for Illumina (New England Biolabs [NEB]) and dual-index NEBNext multiplex oligos (NEB). The library was sequenced on an Illumina MiSeq sequencer using the v3 reagent kit (2 × 300 bp) at the SB RAS Genomics Core Facility (ICBFM SB RAS), generating 371,608 reads. Adapter and low-quality sequences were removed using BBDuk with default parameters (8). Long-read sequencing using a MinION sequencer (Oxford Nanopore, United Kingdom) was done at the All-Russia Research Institute for Agricultural Microbiology (ARRIAM). The SQK-LSK109 kit and the 7th barcode from the EXP-NBD104 kit were used to make the library, omitting the DNA shearing. Guppy_basecaller v. 3.3.0 was used to base call and demultiplex the Nanopore reads.

The output long-read data set (*N*_50_ read length, 1,921 bp) was 1.5 Gb (1.2 · 10^6^ reads). The Flye pipeline v. 2.8-release (9) was used to assemble the Nanopore reads. Final assembly was polished 5 times consecutively using Racon v. 1.3.2 (10) with modifiers (-m 8 -x -6 -g -8 -w 500), followed by a single polish using Medaka v. 1.0.3 (11) with default parameters. Short reads were used to polish the Nanopore assembly with three consecutive runs of Pilon v. 1.22 (12). The calculated coverage was 14× for the Illumina reads and 221× for the Nanopore reads. The 3 contigs correspond to the chromosome (3,610,844 bp; 62.8% GC content) and megaplasmids SMa (1,514,798 bp; 60.6% GC content) and SMb (1,659,814 bp; 62.2% GC content). PGAP v. 4.13 annotation (13) of all three replicons resulted in 3 *rrn-rrl* operons, 54 tRNAs, and 6,166 predicted protein-encoding open reading frames (ORFs). Prophages, genomic islands (GI), and CRISPR/Cas sequences were determined using PHASTER (14), IslandViewer (15), and CRISPR-Cas++ (16), respectively.

Two genomic islands (9 and 11 kb), an intact prophage of 54 kb similar to *Sinorhizobium* phage phiLM21 (GenBank accession number NC_029046), an incomplete prophage of 25 kb similar to *Enterobacteria* phage phi92 (GenBank accession number NC_023693), and 9 potential CRISPR cassettes with one or two spacers and four genes encoding potential Cas proteins (Cas3_0_I) similar to the CRISPR/Cas type I system were found in the genome; similarity was determined using the BLASTn algorithm (17).

### Data availability.

The accession numbers in NCBI are CP065020.1 to CP065022.1 (assemblies), PRJNA678757 (BioProject), and SAMN16812329 (BioSample); SRR13084429 and SRR13084428 are the accession numbers for raw short-read and long-read data in the NCBI SRA. This announcement is for the first version of the S35m genome assembly.
